# Subjective perception of craniofacial growth asymmetries in patients with deformational plagiocephaly

**DOI:** 10.1007/s00784-020-03417-y

**Published:** 2020-07-01

**Authors:** Felix Kunz, Matthias Hirth, Tilmann Schweitzer, Christian Linz, Bernhard Goetz, Angelika Stellzig-Eisenhauer, Kathrin Borchert, Hartmut Böhm

**Affiliations:** 1 Department of Orthodontics, University Hospital Würzburg, Pleicherwall 2, D-97070 Würzburg, Germany; 2 User-centric Analysis of Multimedia Data Group of TU Ilmenau, Ilmenau, Germany; 3 Department of Neurosurgery, University Hospital Würzburg, Würzburg, Germany; 4 Department of Oral and Maxillofacial Surgery, University Hospital Würzburg, Würzburg, Germany; 5 Communication Networks of the University of Würzburg, Würzburg, Germany

**Keywords:** Infants with deformational plagiocephaly (DP), Deformational cranial asymmetry, Subjective perception, Positional skull deformities, Three-dimensional

## Abstract

**Objectives:**

The present investigation aimed to evaluate the subjective perception of deformational cranial asymmetries by different observer groups and to compare these subjective perceptions with objective parameters.

**Materials and methods:**

The 3D datasets of ten infants with different severities of deformational plagiocephaly (DP) were presented to 203 observers, who had been subdivided into five different groups (specialists, pediatricians, medical doctors (not pediatricians), parents of infants with DP, and laypersons). The observers rated their subjective perception of the infants’ cranial asymmetries using a 4-point Likert-type scale. The ratings from the observer groups were compared with one another using a multilevel modelling linear regression analysis and were correlated with four commonly used parameters to objectively quantify the cranial asymmetries.

**Results:**

No significant differences were found between the ratings of the specialists and those of the parents of infants with DP, but both groups provided significantly more asymmetric ratings than did pediatricians, medical doctors, or laypersons. Moreover, the subjective perception of cranial asymmetries correlated significantly with commonly used parameters for objectively quantifying cranial asymmetries.

**Conclusions:**

Our results demonstrate that different observer groups perceive the severity of cranial asymmetries differently. Pediatricians’ more moderate perception of cranial asymmetries may reduce the likelihood of parents to seek therapeutic interventions for their infants. Moreover, we identified some objective symmetry-related parameters that correlated strongly with the observers’ subjective perceptions.

**Clinical relevance:**

Knowledge about these findings is important for clinicians when educating parents of infants with DP about the deformity.

## Introduction

In recent decades, the incidence of deformational plagiocephaly (DP) has increased significantly [1–7], which is assumed to mainly be due to the recommendation for infants to sleep in a supine position in order to reduce the risk of sudden infant death syndrome [8, 9]. As an infant’s head is soft and malleable in the first months of life and is therefore susceptible to deformation [3, 10], this supine sleeping position may result in an asymmetrical occipital flattening of the skull (DP) or—less often—in a symmetrical flattening of the skull (deformational brachycephaly). Characteristic changes to the shape of the head can occur in addition to a unilateral flattening of the occiput, especially in patients with pronounced DP. According to Argenta et al., these changes include an ipsilateral anterior ear shift and an ipsilateral protrusion of the forehead, as well as facial asymmetries [11–14].

Several objective measurements and categorizations exist in the literature for quantifying cranial asymmetries. In 1997, Moss introduced the “Cranial Vault Asymmetry” (CVA), which is defined as the difference between the longest and shortest diagonal of the head [15]. Although the CVA is commonly used and can be easily assessed with a caliper, the disadvantage of this method is that it reduces a three-dimensional asymmetry to a simple two-dimensional measurement [16, 17]. The application of non-invasive 3D stereophotogrammetry allows for capturing a three-dimensional image of an infant’s head as well as possible asymmetries. Modern 3D measurements, such as the “Anterior/Posterior Cranial Asymmetry Index” (ACAI/PCAI, respectively), provide more detailed information about cranial asymmetries [18–20].

Treatment of DP primarily consists of active repositioning as well as of physiotherapy or osteopathy [21]. In patients for whom these conservative therapy approaches have failed to normalize the asymmetry, passive head orthosis can help to guide skull growth in order to correct the deformational asymmetry [22].

Parents of infants with DP are often concerned that their child’s conspicuous appearance may lead to psychological problems, for example, because of bullying in the future [10, 23, 24]. Moreover, they are afraid that the cranial asymmetry might even be recognized by laypersons. Usually, the pediatrician then is the first contact person for parents who are worried about an abnormal shape of their infant’s head. Diagnosis of DP is mainly based on typical clinical symptoms [25] and thus depends on the experience of the pediatrician as well as on his subjective evaluation of the head shape. As DP is considered a primarily cosmetic condition [1, 6, 10, 26], the decision of whether to perform helmet therapy is then mainly based on the subjective perception of the cranial asymmetry by the physician and the parents. Although the subjective perception of cranial asymmetries by different groups of persons seems to play a decisive role in the decision-making process, only very few investigations have thus far focused on it [27, 28].

Therefore, the aim of this prospective cohort study was to assess the subjective perception of deformational cranial asymmetries by parents of infants with DP using a Likert-type scale and to compare these ratings with other observer groups who either form part of infant’s normal social surrounding or have regular contact with infants suffering from DP (such as specialists regularly treating patients with DP, pediatricians, medical doctors, and laypersons). Moreover, this investigation aimed to evaluate which parameter for objectively assessing cranial asymmetries correlates best with the subjective perception of the asymmetry by the observers and to ascertain whether the subjective perception of right-sided posterior cranial asymmetries differs from the subjective perception of left-sided posterior asymmetries. The null hypothesis was that there is no difference in the visual perception of cranial asymmetries between the different observer groups, and there is no correlation between the subjective perception of the asymmetries and the parameters for objectively assessing cranial asymmetries.

## Material and methods

The present study was conceived as an interdisciplinary investigation conducted by the Craniofacial Centre of Würzburg (a team of pediatric neurosurgeons, craniomaxillofacial surgeons, and orthodontists) in cooperation with the Chair of Communication Networks of the University of Würzburg and the User-centric Analysis of Multimedia Data Group of TU Ilmenau. It was conducted in accordance with the ethical guidelines of the Declaration of Helsinki. All parents of the patients gave their written informed consent to participate in the investigation.

### Demonstration examples

In order to assess the subjective perception of positional head asymmetries, a suitable method for demonstrating three-dimensional asymmetries to different observers was necessary. For this purpose, we used standardized video sequences of patients with DP who demonstrated different degrees of severity.

#### Origin of the 3D datasets for the video sequences

From all 3D stereophotogrammetric images of patients with DP that had been taken at the Craniofacial Centre of the University Hospital of Würzburg, we selected 3D datasets that fulfilled the following inclusion and exclusion criteria:

- No head orthosis therapy had previously been conducted
- The age of the infants at the time of the 3D imaging was between 4.5 and 7.5 months
- The cephalic index (width–length ratio) of the patients was between 85 and 95% (typical range for patients with DP)
- The 3D datasets were of good quality

3D datasets of premature infants as well as 3D images of patients with craniosynostosis or any congenital anomalies were excluded. These criteria resulted in a total of 51 datasets of different patients with DP who were potentially eligible as demonstration examples for assessing the subjective perception of cranial asymmetries.

#### Analysis of the 3D datasets for the video sequences

**Fig. 1:**
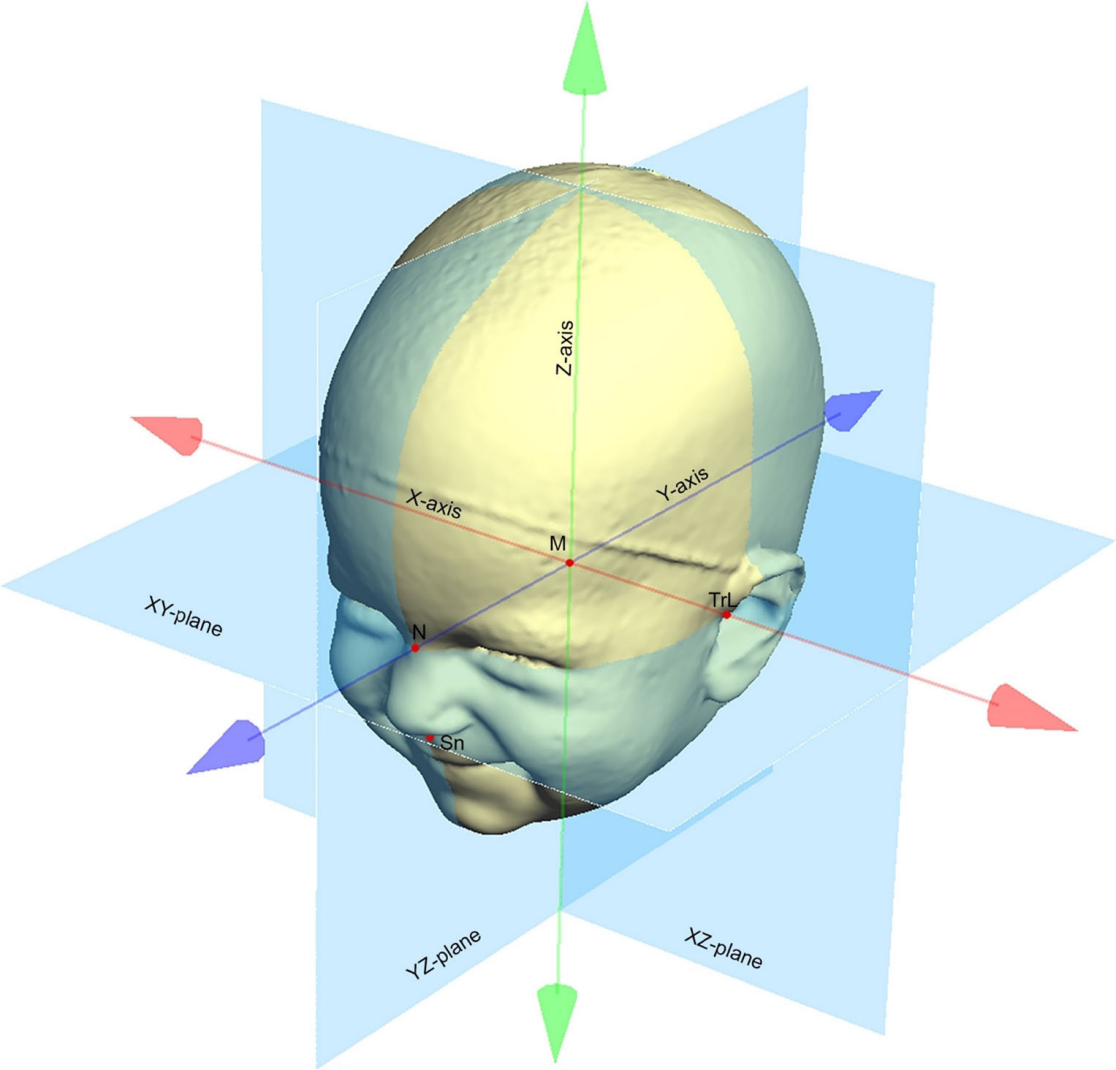
Definition of the coordinate system used to align the 3D stereophotogrammetric datasets of the infants’ heads using the nasion point (N), the subnasal point (Sn), and the tragus points (TrR und TrL) on each side as reference points

**Fig. 2:**
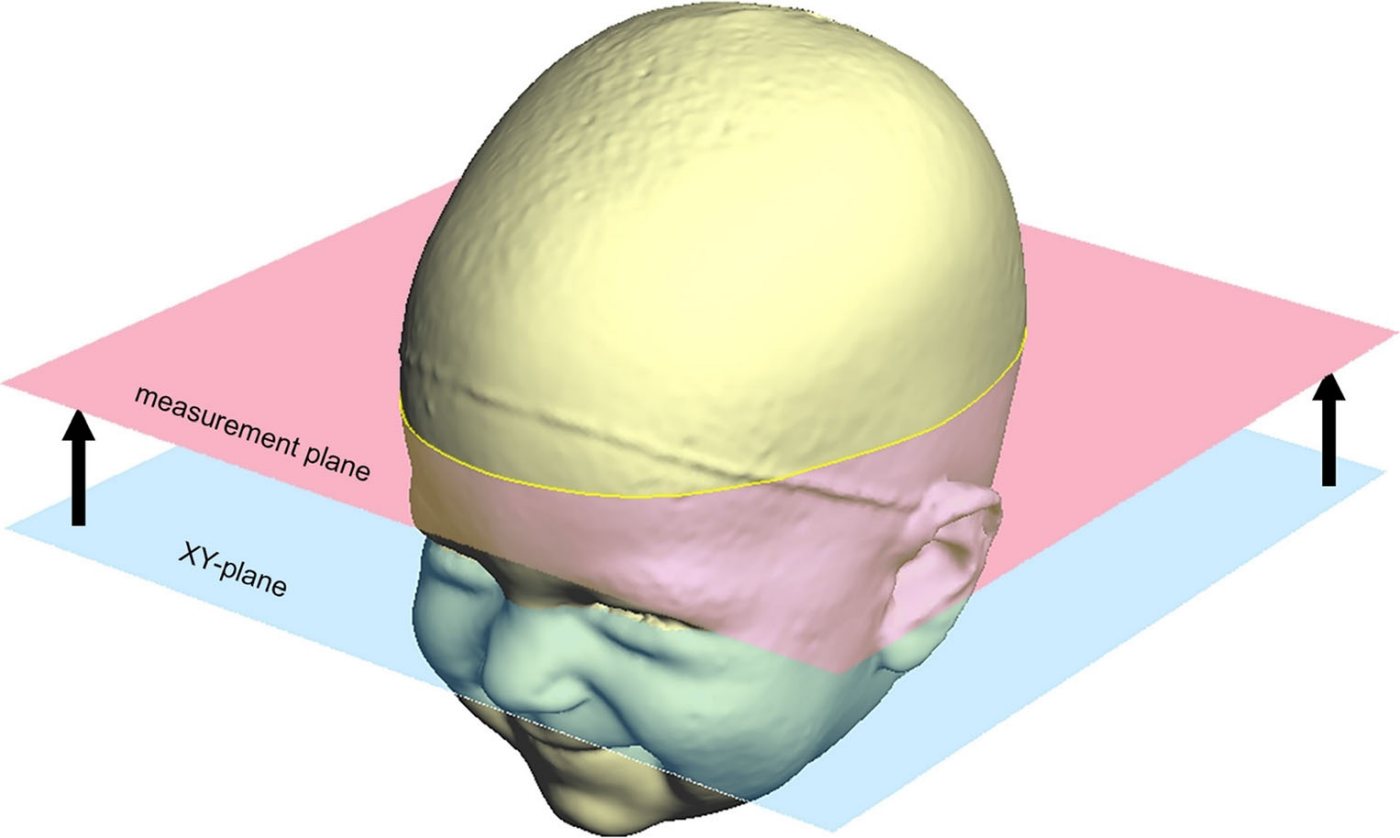
Definition of the measurement plane

**Fig. 3:**
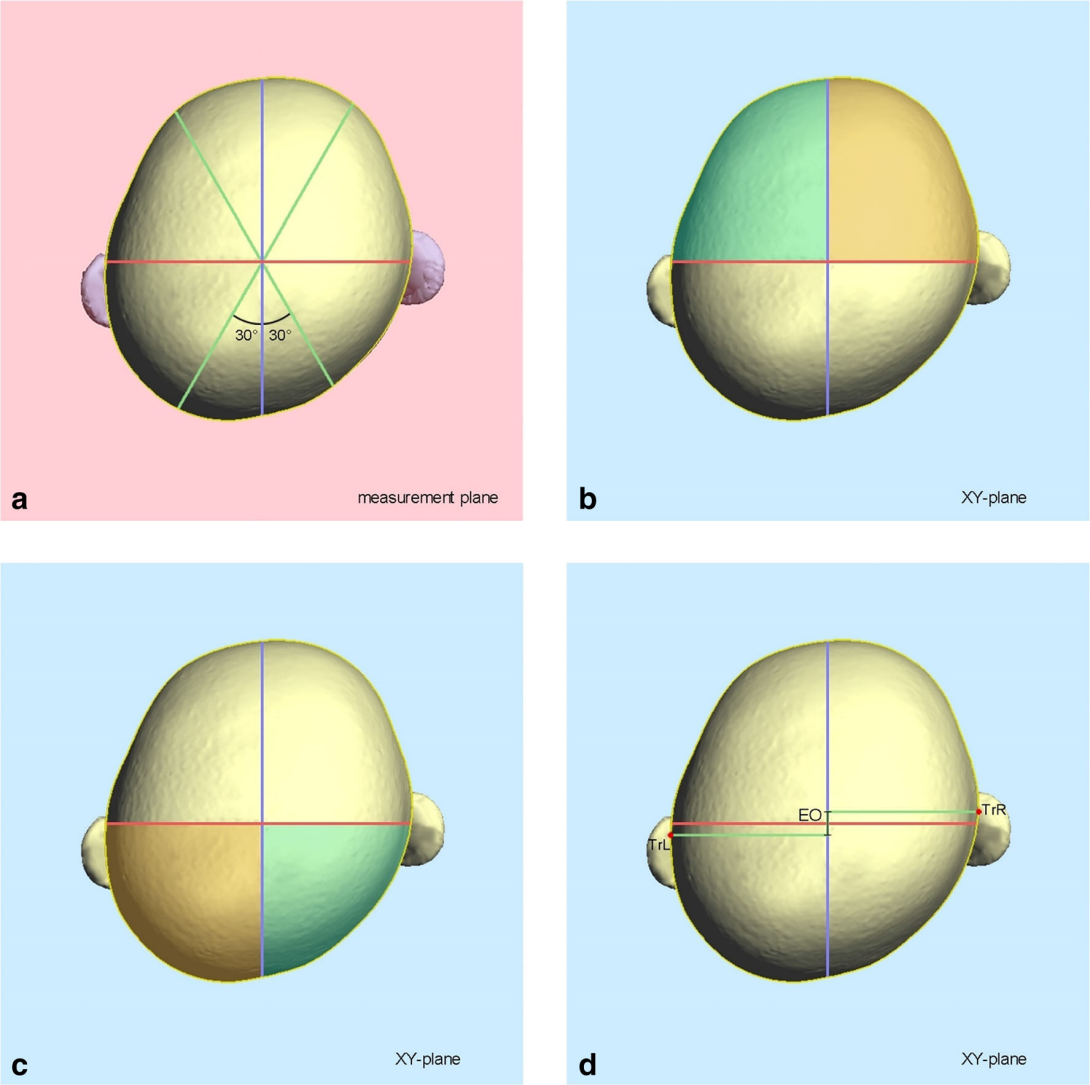
**a**–**d** Definition of the symmetry-related variables for the three-dimensional analysis of the cranial asymmetry. **a** 30° CVA (30° Cranial Vault Asymmetry). **b** ACAI (Anterior Cranial Asymmetry Index). **c** PCAI (Posterior Cranial Asymmetry Index). **d** EO (Ear Offset)

An experienced examiner analyzed the cranial asymmetry of all 51 3D datasets using professional 3D software (Cranioform® Analytics 4.0, Alpnach, Switzerland). First, the stereophotogrammetric images were aligned in a coordinate system using the nasion point (N), the subnasal point (Sn), and the tragus points (TrR und TrL) on each side as reference points. The resulting coordinate system is depicted in Fig. 1. Next, the measurement plane was defined by parallel shifting of the XY-plane of the coordinate system to the maximum length of each infant’s head (Fig. 2). In order to quantitatively assess the cranial asymmetry, we used four symmetry-related variables (Table 1, Fig. 3a–d):

- The “30° Cranial Vault Asymmetry” (30° CVA) is defined as difference between the lengths of the longer and the shorter horizontal diagonal of the infant’s head, measured at a 30° angle to the median-sagittal plane. Even today, this two-dimensional parameter is the gold standard for quantifying cranial asymmetries. This parameter specifies a total value of the asymmetry—therefore, no detailed conclusions can be drawn about the exact area where the asymmetry exists.
- Both the “Anterior Cranial Asymmetry Index” (ACAI) and the “Posterior Cranial Asymmetry Index” (PCAI) are three-dimensional parameters for the analysis of the head shape. For the evaluation, the volume of the neurocranium must first be divided into four volume quadrants. Then, the anterior (ACAI) respectively the posterior (PCAI) volume quadrants of both sides of the head are compared. Using these parameters, the cranial asymmetry can be analyzed separately in the anterior and posterior part of the head (protrusion of the forehead vs. flattening of the occiput).
- The “Ear Offset” (EO) is a parameter for the analysis of the ipsilateral anterior shift of the ear typically occurring in patients with DP. The sagittal discrepancy between the two ears is measured.

**Table 1:** Definition of all symmetry-related variables for the three-dimensional analysis of the cranial asymmetry

Symmetry-related variable	Unit	Definition
30° CVA	cm	Cranial Vault Asymmetry: difference between the lengths of the longer and the shorter diagonal of the infant’s head, measured at a 30° angle to the *Y*-axis at the level of the measurement plane
ACAI PCAI	%	The cranial volume above the XY-plane is divided into four partial volumes by the XZ-plane and YZ-plane of the coordinate system. The Anterior Cranial Asymmetry Index (ACAI) and the Posterior Cranial Asymmetry Index (PCAI) are then calculated using the following equations: $\mathrm{ACAI}=\frac{\left(\mathrm{larger}\ \mathrm{anterior}\ \mathrm{volume}\hbox{--} \mathrm{smaller}\ \mathrm{anterior}\ \mathrm{volume}\right)\times 100}{\mathrm{smaller}\ \mathrm{anterior}\ \mathrm{volume}}$ $\mathrm{PCAI}=\frac{\left(\mathrm{larger}\ \mathrm{posterior}\ \mathrm{volume}\hbox{--} \mathrm{smaller}\ \mathrm{posterior}\ \mathrm{volume}\right)\times 100}{\mathrm{smaller}\ \mathrm{posterior}\ \mathrm{volume}}$
EO	cm	Ear Offset: sagittal offset of the ears, measured at the level of the XY-plane

#### Final selection of the 3D datasets for the video sequences

Of the 51 3D datasets, we selected 10 patients who optimally represented the total cohort in terms of all degrees of severity of positional head asymmetries with respect to all four symmetry-related variables. For this selection process, a “Partitioning Around Medoids” (PAM) cluster analysis was used [29], which enabled the selection of 6 patients who ideally represented all degrees of severity of cranial asymmetries. Based on the suggestions of the medical experts, four additional patients were added to this preselection, resulting in a total of 10 different patients for the evaluation. Table 2 provides an overview of the characteristics of the cranial asymmetries for the final selection of patients for the video sequences.

**Table 2:** Overview of the characteristics of the cranial asymmetry (symmetry-related variables) for the final selection of patients for the video sequences

Patient no.	Symmetry-related variables			
	30° CVA (cm)	ACAI (%)	PCAI (%)	EO (cm)
Patient #1	0.1	5.7	5.0	0.0
Patient #2	0.2	0.8	5.5	0.3
Patient #3	0.4	1.9	8.9	0.3
Patient #4	0.7	2.4	22.6	0.6
Patient #5	0.8	0.3	22.9	0.3
Patient #6	0.8	4.6	9.8	0.2
Patient #7	1.0	5.0	16.8	0.8
Patient #8	1.1	8.4	17.0	0.4
Patient #9	1.3	9.6	19.8	0.5
Patient #10	1.5	5.4	29.7	0.8

Furthermore, all the 3D datasets of these 10 patients were mirrored and added to the final selection, resulting in a total of 20 3D datasets for the assessment of the subjectively perceived cranial asymmetries.

#### Preparation of the video sequences

The video sequences of the 3D datasets of the 10 selected patients were created using the software VAM® (visualization, analysis, and measurement; Version 3.7.6, Vectra, Fairfield, NJ, USA). As the 3D datasets had already been aligned with respect to the coordinate system, this software enabled a precise and standardized definition of different viewing positions for the video sequences.

**Fig. 4:**
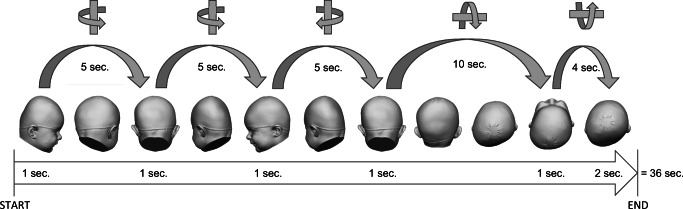
Depiction of the viewpoints used to create the standardized video sequences of the infants’ heads

The video sequences were created using the following viewing positions (Fig. 4):

1. A lateral view of the right side of the infant’s head (1 s)
2. A rotation around the *Z*-axis of the coordinate system up to the posterior view of the infant’s head (5 s)
3. A posterior view of the infant’s head (1 s)
4. A further rotation around the *Z*-axis of the coordinate system up to the lateral view of the left side of the infant’s head (5 s)
5. A lateral view of the left side of the infant’s head (1 s)
6. A rotation around the *Z*-axis of the coordinate system until the posterior view of the infant’s head had been reached (5 s)
7. A posterior view of the infant’s head (1 s)
8. A rotation around the *X*-axis of the coordinate system until the complete prominence of the forehead was visible (10 s)
9. Remaining in this position (1 s)
10. A rotation around the *X*-axis back until the position with a straight view of the XY-plane had been reached (4 s)
11. Remaining in this final position (2 s)

The total length of each video sequence was 36 s.

### Data acquisition

A custom-made web-based presentation platform was used to present the video sequences. This platform enabled us to provide a location-independent survey for assessing the subjective perception of different observers.

The surveys began with a short message to instruct the observers. Afterward, all video sequences were presented in a random order. In addition to the videos of the 20 datasets (10 non-mirrored and 10 mirrored datasets), two randomly selected video sequences (video of patient #1 and video of patient #4) were presented twice to evaluate the reliability of the subjective evaluation of the asymmetry. All observers therefore assessed a total of 22 video sequences within the survey. After watching a video sequence completely at least once, the observers had to evaluate the severity of the subjectively perceived cranial asymmetry of the infants using a 4-point Likert-type scale ranging from “1 = no asymmetry of the head,” “2 = slight asymmetry of the head,” “3 = pronounced asymmetry of the head” to “4 = severe asymmetry of the head.”

After all assessments had been finished, the observers had to answer the following questions:

1. Are you a healthcare professional?
  - If so, what is your specialization?
  - Are you experienced in treating patients with deformational plagiocephaly?
2. Do you have a child or children with deformational plagiocephaly?

Based on their responses to these questions, the observers were subdivided into five different groups:

1. *Specialist group* (e.g., neurosurgeons, craniomaxillofacial surgeons, pediatricians, orthodontists): medical practitioners who treat patients with DP themselves (20 persons)
2. *Pediatrician group*: pediatricians who do not treat patients with DP themselves (32 persons)
3. *Medical doctor group*: other medical doctors (not pediatricians) who do not treat patients with DP themselves (31 persons)
4. *Parent group*: parents of infants with DP (70 persons)
5. *Layperson group*: laypersons who are neither parents of infants with DP nor healthcare professionals (50 persons)

Altogether, 203 observers participated in our investigation. As some observers were not able to assess individual video sequences (52 assessments altogether: 5 at the pediatrician group, 19 at the medical doctor group, 7 at the parent group, and 21 at the layperson group), a total of 4008 assessments (specialist group 400 assessments, pediatrician group 635 assessments, medical doctor group 601 assessments, parent group 1393 assessments, layperson group 979 assessments) were analyzed in this study.

### Statistical analysis

All statistical analyses were performed by a professional statistician (Statworx, Frankfurt, Germany) using the software IBM®, SPSS®, Statistics Version 25.0 for Windows (IBM, Ehningen, Germany).

To determine the reliability of the subjective assessments of the cranial asymmetries made by the observers, we set up cross tables for the two datasets which had been presented twice to all observers and calculated the γ-coefficient. For all statistical analyses, we assumed that the assessment of the subjective perception of the asymmetry (using a 4-point Likert-type scale) was a metric variable. The subjective perceptions of the cranial asymmetries by the different observer groups were compared using a multilevel modelling linear regression analysis. For this analysis, the parent group was set as the reference category as the parents’ perception of the asymmetry was assumed to usually be the main reason for seeking therapy. In a second step, we compared all observer groups with one another using *t* tests with Bonferroni correction as form of a post hoc analysis in order to further examine group differences.

We used Spearman’s rank correlation coefficient in the statistical evaluation of which parameter for objectively assessing the cranial asymmetry correlated best with the subjective perception of the asymmetry. The comparison of the subjective perception of right-sided and left-sided posterior cranial asymmetries was analyzed using *t* tests for paired samples. As patient #1 and patient #2 demonstrated a 30° CVA of less than 3 mm and therefore did not fulfill the criteria for DP according to Moss or Mortenson et al. [15, 16], the comparison of the subjective perception of a right-sided and a left-sided posterior cranial asymmetry was not suitable. Consequently, we excluded these two patients from this analysis.

The significance level was 5% for all procedures.

## Results

### Reliability of the assessments of the asymmetry

High reliability was proven for the subjective assessments of the cranial asymmetries (Table 3). For the two datasets (which were presented twice to the observers), 70.3% and 70.8%, respectively, of the observers rated the degree of asymmetry evenly for both assessments on the 4-point Likert-type scale. This concordance of ratings is also represented with high γ-coefficients of .69 and .81 (with *p* < .001) for both datasets, respectively.

**Table 3:** Reliability of the assessments of the subjective perception made by the observers. The cross tables depict the first and the second assessment of the datasets, which were evaluated twice by all observers

	**Patient 1**		**Second assessment**						
			**1**	**2**	**3**	**4**	**γ**	***p***	**Perfect match**
	**First assessment**	**1**	**114**	37	1	0	.69	< .001**	70.3%
		**2**	18	**27**	3	0			
		**3**	0	1	**0**	0			
		**4**	0	0	0	**1**			
	**Patient 4**		**Second assessment**						
			**1**	**2**	**3**	**4**	**γ**	***p***	**Perfect match**
	**First assessment**	**1**	**6**	8	0	0	.81	< .001**	70.8%
		**2**	6	**108**	18	1			
		**3**	0	20	**29**	1			
		**4**	0	2	3	**0**			

The bold numbers highlight the matches of both ratings

γ=γ-coefficient, *p p* value

*Significance for *p* < .05; **significance for *p* < .01

### Descriptive results

The descriptive results are depicted in Table 4.

**Table 4:** Mean values of the subjectively perceived severity of the cranial asymmetry of each patient by the different observer groups, evaluated using the 4-point Likert-type scale ranging from 1 (“no asymmetry of the head”) to 4 (“severe asymmetry of the head”)

Patient no.	All observers (*N* = 203)	Specialist group (*N* = 20)	Pediatrician group (*N* = 32)	Medical doctor group (*N* = 31)	Parent group (*N* = 70)	Layperson group (*N* = 50)
Patient #1	1.30	1.25	1.21	1.31	1.32	1.33
Patient #2	1.89	1.85	1.76	1.76	2.00	1.89
Patient #3	1.68	1.80	1.69	1.80	1.64	1.61
Patient #4	2.23	2.53	2.30	2.12	2.29	2.04
Patient #5	3.23	3.48	3.08	3.00	3.44	3.10
Patient #6	1.92	1.98	1.94	1.92	1.96	1.82
Patient #7	2.47	2.63	2.32	2.41	2.59	2.39
Patient #8	2.95	3.15	2.83	2.74	3.17	2.75
Patient #9	3.05	3.38	2.92	2.84	3.17	2.97
Patient #10	3.03	3.40	3.14	2.77	3.14	2.82
Average of all patients	2.38	2.54	2.32	2.27	2.47	2.27

We acquired a total of 203 observers for the subjective assessment of cranial asymmetries (57% female, 40% male, 3% unspecified). The average subjective assessment of the cranial asymmetry for all 20 datasets on the 4-point Likert-type scale was *M* = 2.38 (*SD* = 0.90).

The observers were subdivided into five groups. The cranial asymmetries were perceived as being most asymmetric by the specialist group (35% female, 60% male, 5% unspecified), with an average rating of *M* = 2.54 (*SD* = 0.92), followed by the parent group (74% female, 22% male, 4% unspecified), with an average rating of *M* = 2.47 (*SD* = 0.93). The pediatrician group (22% female, 78% male), the layperson group (60% female, 36% male, 4% unspecified), and the medical doctor group (61% female, 39% male) subjectively assessed the shape of the head as being less asymmetric, with an average rating of *M* = 2.32 (*SD* = 0.84), *M* = 2.27 (*SD* = 0.87), and *M* = 2.27 (*SD* = 0.85), respectively.

### Comparison between the different observer groups

The detailed results of the comparison of the subjective asymmetry assessments between the different observer groups are depicted in Tables 5 and 6.

**Table 5:** Linear regression analysis (multilevel modelling) to compare the ratings of the subjective perception of the cranial asymmetry made the different observer groups. The parent group was set as the reference category

		*95% confidence interval*					
Parameter	*B*	*Lower limit*	*Upper limit*	*SE*	*df*	*t*	*p*
Constant	2.39	2.35	2.43	0.02	3181.86	110.53	<.001**
Parent group vs. specialist group	0.08	− 0.01	0.17	0.46	3174.09	1.73	.084
Parent group vs. pediatrician group	− 0.13	− 0.21	− 0.06	0.04	3187.27	− 3.47	< .001**
Parent group vs. medical doctor group	− 0.19	− 0.26	− 0.11	0.03	3188.14	− 4.64	< .001**
Parent group vs. layperson group	− 0.19	− 0.26	− 0.13	0.03	3188.14	− 5.68	< .001**

*B* regression coefficient, *SE* standard error, *df* degrees of freedom, *t t* value, *p p* value

*Significance for *p* < .05; **significance for *p* < .01

**Table 6:** Comparison of all observer groups with one another using *t* tests with Bonferroni correction as a post hoc analysis

			*95% confidence interval*		
Observer group 1 vs. group 2		Difference between both groups	*Lower limit*	*Upper limit*	*p*
Specialist group	Pediatrician group	0.213	0.069	0.358	< .001**
	Medical doctor group	0.262	0.116	0.408	< .001**
	Parent group	0.079	− 0.050	0.208	.843
	Layperson group	0.270	0.136	0.405	< .001**
Pediatrician group	Medical doctor group	0.049	− 0.080	0.178	> .999
	Parent group	− 0.134	− 0.243	− 0.026	.005**
	Layperson group	0.057	− 0.059	0.173	> .999
Medical doctor group	Parent group	− 0.183	− 0.294	− 0.072	< .000**
	Layperson group	0.008	− 0.109	0.126	> .999
Parent group	Layperson group	0.191	0.097	0.286	< .001**

*p p* value

*Significance for *p* < .05; **significance for *p* < .01

The subjective assessments of the cranial asymmetries by the specialist group were significantly higher compared with those of all other groups with the exception of the parent group. The assessments made by the observers of the pediatrician group were similar to those made by the medical doctor group and to those of the layperson group yet were significantly lower compared with those of the specialist group and of the parent group. The medical doctor group rated the asymmetry similarly to the pediatrician group and the layperson group yet rated it significantly lower than did the specialist group and the parent group. The ratings made by the parent group were significantly higher compared with those of the pediatrician group and of the medical doctor group, but there was no significant difference compared with the ratings of the specialist group.

### Correlation between the subjective perception and the symmetry-related variables of the cranial asymmetry

The detailed results of the correlations between the subjective perception and the symmetry-related variables of the cranial asymmetry are depicted in Table 7.

**Table 7:** Correlation between the subjective perception and the symmetry-related variables of the cranial asymmetry using Spearman’s rank correlation coefficient

		30° CVA		ACAI		PCAI		EO	
Observer group	*N*	*rho*	*p*	*rho*	*p*	*rho*	*p*	*rho*	*p*
All observers	203	.80	< .001**	.13	.577	.87	< .001**	.56	.010*
Specialist group	20	.83	< .001**	.16	.500	.90	< .001**	.59	.007**
Pediatrician group	32	.85	< .001**	.17	.484	.93	< .001**	.62	.003**
Medical doctor group	31	.80	< .001**	.15	.516	.85	< .001**	.54	.014*
Parent group	70	.76	< .001**	.11	.639	.83	< .001**	.55	.012*
Layperson group	50	.77	< .001**	.15	.542	.83	< .001**	.54	.015*

*rho* Spearman’s rank correlation coefficient, *p p* value

*Significance for *p* < .05; **significance for *p* < .01

There were statistically significant correlations between the subjective perception of the observers and the 30° CVA, the PCAI, and the EO. The highest Spearman’s rho correlation coefficients were observed for the PCAI, followed by the 30° CVA and finally by the EO. In contrast, the ACAI did not significantly correlate with the subjective perception of the observers.

The Spearman’s rho correlation coefficients of the different observer groups were very similar, although the pediatrician group and the specialist group demonstrated the highest correlation coefficients, while the parent group and the layperson group demonstrated the lowest correlation coefficients.

### Influence of the side of the cranial asymmetry on the subjective perception

The analysis of the influence of the side of the cranial asymmetry on the subjective perception of the observers is depicted in Table 8.

**Table 8:** Analysis of the influence of the side of the cranial asymmetry on the subjective perception of the observers using *t* tests for paired samples

	Descriptive analysis *M* (*SD*)		*t* test
Patient no.	Right-sided post. asymmetry	Left-sided post. asymmetry	*p*
Patient #3	1.70 (0.59)	1.67 (0.55)	.623
Patient #4	2.24 (0.61)	2.23 (0.61)	.844
Patient #5	3.23 (0.65)	3.23 (0.70)	.924
Patient #6	1.95 (0.53)	1.89 (0.59)	.207
Patient #7	2.54 (0.65)	2.42 (0.60)	.019*
Patient #8	2.98 (0.70)	2.91 (0.70)	.220
Patient #9	3.07 (0.66)	3.02 (0.69)	.319
Patient #10	3.04 (0.70)	3.01 (0.70)	.605

*M* mean, *SD* standard deviation, *p p* value

*Significance for *p* < .05; **significance for *p* < .01

In seven out of eight patients, there was no difference in the subjective perception of the cranial asymmetry when comparing right-sided and left-sided posterior asymmetries. Only the assessments of patient #7 demonstrated a side-specific difference in the subjective perception of the cranial asymmetry.

## Discussion

Positional head asymmetries are the most common skull deformities in infancy [7, 30–32]. Although DP is considered mainly a cosmetic condition [1, 6, 10, 26], parents of infants with DP are often concerned that their child’s conspicuous appearance may lead to psychological problems [10, 23, 24]. Thus far, only very few studies have investigated the subjective perception of deformational skull asymmetries [27, 28]. The aim of the present investigation was therefore to assess the subjective perception of deformational cranial asymmetries by different observer groups and to correlate the subjective perception with objective parameters in order to quantify the asymmetry.

For this purpose, we selected all 3D stereophotogrammetric datasets of patients with DP who had consulted the Craniofacial Centre of the University Hospital of Würzburg and who fulfilled a variety of requirements. Afterward, all preselected 3D datasets were aligned to a coordinate system, and four different symmetry-related variables were analyzed. In several previous publications, we could already prove that this method for the analysis of cranial asymmetries is very reliable [18–20, 33]. However, automated identification of the landmarks required to align the datasets might possibly further improve the reliability of the three-dimensional cranial measurements. In this context, the procedure presented by Lippold et al. could be an appropriate approach [34]. Of all these 3D datasets, we used a PAM cluster analysis to select ten patients who represented all degrees of severity of deformational cranial asymmetries with respect to all of the four symmetry-related variables. Referring to the classifications made by Moss and Mortenson et al., this final sample contained 3D datasets of all degrees of severity of deformational head asymmetries, ranging from almost-perfect symmetry to severe deformational skull asymmetries [15, 16].

In a next step, we created standardized video sequences that showed the different facets of the cranial asymmetries of these ten patients from lateral, posterior, and cranial viewpoints. In collaboration with the Chair of Communication Networks of the University of Würzburg and the User-centric Analysis of Multimedia Data Group of TU Ilmenau, a web-based presentation platform was established to present these videos location-independently to different observers. These observers subjectively rated the severity of the cranial asymmetries using a 4-point Likert-type scale. To prove the reliability of this method, all test persons unknowingly rated two of the videos twice. The cross tables demonstrated high reliability for the subjective assessments, with a perfect match rate (if a test person rated the same videos equally) of about 70%. This method was therefore deemed suitable for reliably assessing the subjective perception of the observers. One strength of the present investigation was the presentation of a mirrored counterpart of every 3D dataset of the infants with DP to the observers. By so doing, we were able to rule out side-specific biases. It also allowed us to show that the side of the posterior asymmetry seems not to affect the visual perception of cranial asymmetries.

One of the main aims of the present investigation was to assess the subjective perception of cranial asymmetries by different observer groups and to verify whether these groups perceive cranial asymmetries differently. To this end, we specifically acquired 203 observers who either formed part of infant’s normal social surrounding or had regular contact with infants suffering from DP. The cohort of observers was subdivided into five groups: specialists, pediatricians, medical doctors, parents of infants with DP, and laypersons.

Our results reveal that there was no significant difference in the subjective perception of the severity of the cranial asymmetry when comparing the results for the specialists and the parent group or when comparing the results of the pediatricians, the medical doctors, and the laypersons. However, the ratings of the perceived asymmetry as assessed by the specialists and by the parent group were significantly more asymmetric as compared with those of all other groups. This finding might be explained by the fact that both specialists and parents of infants with DP have experience with the appearance of cranial asymmetry and are thus primed to notice it. These groups may consequently also be highly sensitive to very minor cranial asymmetries [35].

On the other hand, pediatricians perceive cranial asymmetries more moderately than do specialists or the parents of patients with DP, which may lead to problems in the management of patients with DP. Usually, the pediatrician is either the first to diagnose DP or the first contact person for parents who are worried about an abnormal shape of their infant’s head. The recommendations of the pediatrician are known to influence decision-making, for example, regarding the optimal therapy approach [36, 37]. The fact that pediatricians perceive cranial asymmetries more moderately than do specialists or parents of infants with DP may result in an insufficient motivation on the part of the parents in seeking therapeutic interventions for their children and may therefore also result in delayed therapy or a compromised treatment outcome [18, 31, 38, 39]. Due to the study design, however, it cannot be assessed whether the statistically significant differences in the perception of cranial asymmetries between the different observer groups are also clinically relevant, as the differences determined on the Likert-type scale between some groups were only very small. This limitation must be taken into account when interpreting the results discussed above. Thus, for example it could also be argued that the slightly different assessment by pediatricians will probably not prevent them from referring patients to a specialist at least for a detailed diagnosis, especially if the parents insist on it.

Furthermore, the fact that parents of infants with DP assess cranial asymmetries as being more asymmetric than do laypersons is critical for practitioners who treat infants with DP. Although there are several investigations that demonstrate that most parents are very satisfied with the outcomes of the therapeutic interventions, some parents are still concerned about possible bullying in the future because of their child’s appearance due to a remaining asymmetry of the head after therapy [10, 24, 35, 40, 41]. In these situations, the practitioner can comfort the parents by relaying the fact that laypersons will likely not notice this remaining asymmetry as well as do the parents.

Another major aim of the present investigation was to evaluate which parameter for the objective assessment of cranial asymmetry correlates best with the subjective perception of the asymmetry. Our results demonstrate that the subjective perception of the observers correlated significantly with the objective symmetry-related variables 30° CVA, PCAI, and EO, whereas it did not correlate with the ACAI. These results are of particular interest as the ACAI, the PCAI, and the EO quantify specific parts of cranial asymmetry. Our results thus reveal that the subjective perception of cranial asymmetry correlates best with the posterior flattening of the skull (PCAI), followed by the anterior shift of the ear (EO), whereas the protrusion of the forehead (ACAI) seems not to be perceived as critically. The two-dimensional 30° CVA (the gold standard for quantifying cranial asymmetries) independently demonstrated very high degrees of correlation with the subjective perception of the observers and was only outperformed by the three-dimensional PCAI.

All the results mentioned above were very similar for the total cohort of observers and for all subgroups. This result is only partially in line with the findings of Feijen et al., who were only able to prove significant correlations between objective parameters for the cranial asymmetries and the subjective perception of physicians but not for parents of infants with DP [1]. This contradiction with our results may be explained by the fact that in the investigation by Feijen et al., the parents only rated the asymmetry of their own infants, which may have resulted in a psychologically biased assessment.

In this context, the results of the present study should also be discussed in detail. As described above, specialists and parents of infants with DP subjectively rated the severity of cranial asymmetry very similarly. Surprisingly, the parents of affected children did not demonstrate such high correlations of the subjective perception of the asymmetries with the objective parameters compared with the specialists. We therefore assume that parents, like specialists, are very sensitive to head asymmetries and generally tend to classify them as severe, but that parents are less able to differentiate finer gradations of cranial asymmetry than specialists. In this respect, pediatricians were also able to differentiate more sensitively between different degrees of cranial asymmetries.

## Conclusion

The present investigation analyzed many aspects of the subjective perception of cranial asymmetries in infants with DP for the first time. We were able to demonstrate that specialists, pediatricians, medical doctors, parents of infants with DP, and laypersons perceive the severity of cranial asymmetries differently. Both specialists and parents of infants with DP perceived the cranial deformities as being significantly more asymmetric than did the remaining groups (e.g., the pediatrician group). As pediatricians are usually the first contact persons for parents worried about an abnormal shape of their infant’s head, this more moderate perception of the cranial asymmetry by the pediatrician may result in insufficient motivation on the part of the parents to seek therapeutic interventions and therefore also in a compromised treatment outcome. Additionally, our results reveal that some objective symmetry-related parameters strongly correlate with the subjective perception of our observers, especially the PCAI as a three-dimensional parameter for quantifying the posterior flattening of the skull. On the other hand, the side of the posterior asymmetry seems not to affect the visual perception of the asymmetry. These facts are relevant and critical for clinicians and pediatricians when comprehensively educating parents of infants with DP about the deformity.
